# Child contact management in high tuberculosis burden countries: A mixed-methods systematic review

**DOI:** 10.1371/journal.pone.0182185

**Published:** 2017-08-01

**Authors:** Daria Szkwarko, Yael Hirsch-Moverman, Lienki Du Plessis, Karen Du Preez, Catherine Carr, Anna M. Mandalakas

**Affiliations:** 1 Department of Family Medicine and Community Health, The University of Massachusetts Medical School, Worcester, Massachusetts, United States of America; 2 Department of Family Medicine, Alpert Medical School of Brown University, Providence, Rhode Island, United States of America; 3 ICAP at Columbia University, Mailman School of Public Health, New York, New York, United States of America; 4 Department of Epidemiology, Columbia University, New York, New York, United States of America; 5 Desmond Tutu TB Centre, Department of Paediatrics and Child Health, Faculty of Medicine and Health Sciences, Stellenbosch University, Tygerberg, South Africa; 6 Lamar Soutter Library, The University of Massachusetts Medical School, Worcester, Massachusetts, United States of America; 7 Global TB Program, Department of Pediatrics, Baylor College of Medicine, Houston, Texas, United States of America; Médecins Sans Frontières (MSF), SOUTH AFRICA

## Abstract

Tuberculosis (TB) remains a leading cause of morbidity and mortality worldwide. Considering the World Health Organization recommendation to implement child contact management (CCM) for TB, we conducted a mixed-methods systematic review to summarize CCM implementation, challenges, predictors, and recommendations. We searched the electronic databases of PubMed/MEDLINE, Scopus, and Web of Science for studies published between 1996–2017 that reported CCM data from high TB-burden countries. Protocol details for this systematic review were registered on PROSPERO: International prospective register of systematic reviews (#CRD42016038105). We formulated a search strategy to identify all available studies, published in English that specifically targeted a) population: child contacts (<15 years) exposed to TB in the household from programmatic settings in high burden countries (HBCs), b) interventions: CCM strategies implemented within the CCM cascade, c) comparisons: CCM strategies studied and compared in HBCs, and d) outcomes: monitoring and evaluation of CCM outcomes reported in the literature for each CCM cascade step. We included any quantitative, qualitative, mixed-methods study design except for randomized-controlled trials, editorials or commentaries. Thirty-seven studies were reviewed. Child contact losses varied greatly for screening, isoniazid preventive therapy initiation, and completion. CCM challenges included: infrastructure, knowledge, attitudes, stigma, access, competing priorities, and treatment. CCM recommendations included: health system strengthening, health education, and improved preventive therapy. Identified predictors included: index case and clinic characteristics, perceptions of barriers and risk, costs, and treatment characteristics. CCM lacks standardization resulting in common challenges and losses throughout the CCM cascade. Prioritization of a CCM-friendly healthcare environment with improved CCM processes and tools; health education; and active, evidence-based strategies can decrease barriers. A focused approach toward every aspect of the CCM cascade will likely diminish losses throughout the CCM cascade and ultimately decrease TB related morbidity and mortality in children.

## Background

Tuberculosis (TB) remains a leading infectious cause of morbidity and mortality worldwide. In 2015, there were an estimated 10.4 million incident cases of TB and 1.8 million TB deaths. At least 1.0 million (10%) of these cases were estimated to be in children [1]. Children <15 years contribute approximately 10–20% of disease burden in TB-endemic areas [2]. The risk of TB disease progression in children is significantly higher than in adults, particularly in children <5 years [3, 4]. Additionally, there is increased risk of TB during adolescence [5, 6], which may result from new infection or progression of latent TB infection to active disease. Although young children are at great risk of progressing to severe disease and death [4, 7], isoniazid preventive therapy (IPT) decreases the progression of TB disease by 59% in this vulnerable population [8].

In 2006, the World Health Organization (WHO) published guidelines for the management of child TB that urged national TB control programs (NTPs) to (1) conduct contact investigations and offer IPT in child contacts <5 years and HIV-positive contacts of any age and (2) screen symptomatic child contacts 5–14 years for TB disease [9–11]. Given the significant burden of TB disease in young children, and the continued challenges in child TB diagnostics, successful implementation of child contact management (CCM) is an important upstream strategy to prevent TB in children. CCM is also an opportunity for early case detection and treatment [12]. Many NTPs have adopted these guidelines; however, implementation in high burden countries (HBCs) remains low with many countries experiencing operational challenges in CCM [13]. These challenges result in poor IPT initiation and completion rates.

CCM includes identifying, screening, and evaluating child contacts exposed to bacteriologically-positive TB, as well as initiating and completing either preventive therapy or appropriate treatment for active TB. These CCM steps mirror the steps of the HIV care cascade steps, which include identifying individuals at high risk, testing, initiating treatment, and retaining individuals in care [14, 15]. A clear understanding of the steps at which individuals are lost and the barriers that impact each step has helped to optimize HIV prevention strategies and is the cornerstone of initiatives such as the 2013 HIV Care Continuum Initiative in the United States [16]. A recent systematic review and meta-analysis by Alsdurf and colleagues proposed the application of the HIV care framework to latent TB care for all ages to identify barriers and improve steps in the latent TB care cascade [17]. The results demonstrated that individuals were lost at every step of this cascade. Nevertheless, this review did not address the unique challenges of preventing child TB in HBCs.

### Child contact management cascade

**Fig 1:**
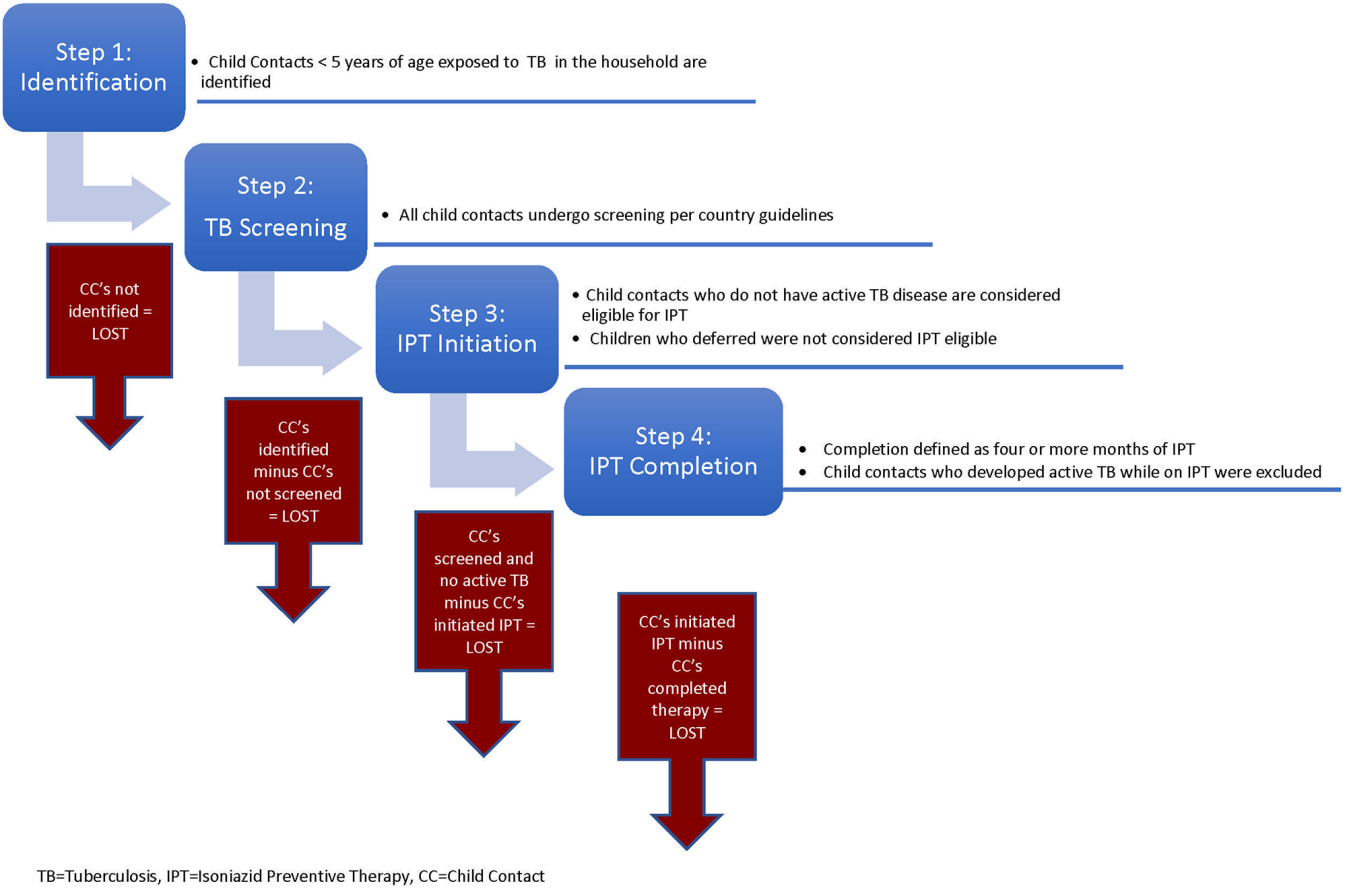
The child contact management cascade for preventive therapy.

In line with the first two pillars of the End TB Strategy that recommend: (1) integrated, patient-centered TB care and prevention and (2) bold policies and supportive systems, we propose a CCM care cascade for child contacts <5 years who have been exposed to TB in the household [18, 19]. Household definition varies among programs, however, a common definition used in country demographic health surveys and CCM programs is, ‘a person or group of people, related or unrelated to each other, who live together in the same dwelling unit and share a common source of food [20, 21].’ Per WHO recommendations, the cascade starts with the *identification* of all exposed child contacts aged <15 years. The first loss occurs if identification of child contacts is incomplete. In the second step, all child contacts <5 years and symptomatic or HIV-positive contacts 5–14 years undergo *screening* and evaluation to rule out active TB. The second possible loss of child contacts occurs at this step. The third cascade step includes *IPT initiation*, which only applies to child contacts <5 years or HIV-positive children. We assume IPT eligibility for all child contacts who were screened and did not have TB disease. The final step in the cascade is *IPT completion*. The CCM cascade for child contacts for preventive therapy, i.e., in child contacts <5 years is summarized in Fig 1.

For the first time, the 2016 WHO global tuberculosis report incorporated measures of reported rates of IPT initiation in child contacts <5 years [1]. Only nine of 30 HBCs reported data for this new indicator. From the data available, an estimated 7.1% of eligible child contacts initiated IPT, far below the 90% target goal [1]. This further highlights the need to employ a standardized care cascade to CCM to better understand the existing gaps and barriers, and devise strategies to improve outcomes. Additionally, programmatic strategies and challenges as well as perceptions from healthcare workers (HCWs) and family members regarding CCM are important considerations to NTPs conducting CCM. We therefore conducted a mixed-methods systematic review of evidence available from HBCs to (1) apply a care cascade to CCM and report on child contact losses at each CCM cascade step; (2) summarize common challenges, implementation gaps, and predictors of outcomes within CCM programs; and (3) identify CCM strategies implemented at each step in the cascade. The goal of this review is to provide guidance to NTPs that are establishing or improving their CCM initiatives.

## Materials and methods

### Search strategy and selection criteria

We searched the electronic databases of PubMed/MEDLINE (NCBI), Scopus (Elsevier), and Web of Science (Thomson Reuters) for studies published between January 1, 1996 and May 23, 2017. The search strategy was designed with a librarian (CC) to identify studies from HBCs that describe or analyze CCM strategies throughout the CCM cascade steps (identification, screening, IPT initiation, and IPT completion). This time period was selected so that we could assess progress of CCM 10 years pre- and post-WHO 2006 CCM guidelines. For initial search terms, we used the following free text and MeSH terms: tuberculosis, isoniazid preventive therapy, contact management, child, and a list of countries with an estimated TB incidence of greater than 40:100,000 population during 2015, to ensure inclusion of all countries listed on the WHO TB, TB/HIV and MDR TB high burden lists [1]. The exact and detailed search strategies, including related terms, are reported in S1 File. We followed the Preferred Reporting Items for Systematic Reviews and Meta-Analyses (PRISMA) guidelines to structure our systematic review preparation and reporting (S2 File) [22]. Details of the protocol for this systematic review were registered on PROSPERO: International prospective register of systematic reviews (#CRD42016038105).

We included manuscripts describing countries with TB incidence >100:100,000 to capture a homogenous set of countries and support more rigorous comparisons and program evaluations. We therefore formulated a final search strategy to identify all available studies, published in English that specifically targeted a) population: child contacts (<15 years) exposed to TB in the household, from programmatic settings in HBCs, defined as TB incidence >100:100,000, b) interventions: CCM strategies implemented within the CCM cascade, c) comparisons: CCM strategies studied and compared in HBCs, and d) outcomes: monitoring and evaluation of child contact outcomes reported in the literature for each CCM cascade step. We included any quantitative (cohort, case control, cross sectional, ecological studies), qualitative, mixed-methods study design except for randomized-controlled trials, editorials or commentaries. Initial search results were imported into EndNote, where duplicates were removed. Two authors (DS, YHM) independently screened the titles and abstracts to determine each study's initial eligibility for inclusion. Disagreements between authors during screening were resolved through discussion with the remaining authors. Five authors (DS, YHM, LDP, KDP, AMM) divided the included studies and independently conducted full text review to determine each study’s eligibility for final inclusion. Discrepancies about study eligibility were discussed and resolved through consensus. Studies not meeting these criteria were excluded. Studies were also excluded if they included only adult contacts, were non-human studies, were related solely to drug resistant CCM, or involved IPT in HIV-positive children with no known TB exposure. Given the heterogeneity of the studies conducted in programmatic settings, we could not conduct a meta-analysis.

### Data extraction

Two authors (DS, YHM) performed the full data extraction. Data were extracted using a standardized data extraction tool created in Google Forms that was pre-piloted by authors. Data auto filled into a Google worksheet that was exported into Microsoft Excel 2008. Data extracted included article title, authors, year published, study year, journal title, study aims, study design, methodology, phase of CCM addressed or reported (identification, screening, IPT initiation, IPT completion), CCM challenges, CCM recommendations, and predictors for the CCM cascade. We extracted both quantitative and qualitative data.

#### Quantitative data

Quantitative data was collected when available to highlight the child contact losses at each step and calculate the proportion of child contacts completing each CCM cascade step. Data included the number of index cases, child contacts <5 years identified, child contacts <5 years screened for TB disease, child contacts <5 years eligible for IPT, child contacts <5 years initiating IPT, and child contacts <5 years completing IPT. We defined child contacts screened as the number of child contacts who completed evaluation to rule out active TB disease. Screening varied from simple symptom-based screening to protocols that incorporate tuberculin skin testing, chest x-ray, and other diagnostic tests such as evaluation of gastric aspirate. Child contact losses at this step were measured by subtracting child contacts screened from those identified. The proportion of child contacts screened was defined as the number of child contacts screened of those who were identified. IPT eligibility was defined as child contacts who completed screening and did not have active TB disease. If authors reported deferral reasons (e.g. previously treated with IPT, already treated for active TB disease), these child contacts were not considered IPT eligible. Therefore, losses were measured by subtracting child contacts who initiated IPT from those who were screened and did not have a deferral reason. The proportion of child contacts who initiated IPT was defined as the number of child contacts who initiated IPT of those who were IPT eligible. In cases where the number of child contacts eligible for IPT was not available, we used the number of child contacts screened as the denominator for the IPT initiation rate. IPT completion losses were measured by subtracting child contacts who completed IPT from those who initiated IPT. The proportion of child contacts who completed IPT was defined as the number of child contacts who completed at least 4/6 months of IPT of those who initiated IPT. Outcomes regarding predictors for completion of CCM cascade steps for contacts <15 years were also collected. Two authors (DS, YHM) compiled the quantitative data and performed descriptive statistics using Microsoft Excel.

#### Qualitative data

Qualitative data, including findings from in-depth interviews, focus group discussions, and study authors’ observations, were systematically extracted. Data included CCM interventions, CCM strategies, CCM challenges, and CCM recommendations as described by authors, HCWs, or family members. Interventions, strategies, and challenges were grouped into broad categories. Initial codes were independently generated from a review of Assefa and colleagues [23] by two reviewers (DS and YHM) and cross-checked to enhance consensus on primary codes. Reviewers (DS and YHM) then used an iterative process to further refine and contextualize codes through constant and discursive comparative analysis [24, 25] within and across studies to facilitate a comprehensive understanding of CCM challenges and recommendations. Once concordance on codes was achieved, all studies were coded. Finally, reviewers (DS, YHM, LDP, KDP, AMM) worked together to summarize the codes and finalize seventeen sub-themes into seven meaningful themes that related to the review aims.

## Results

### Initial screening protocol

**Fig 2:**
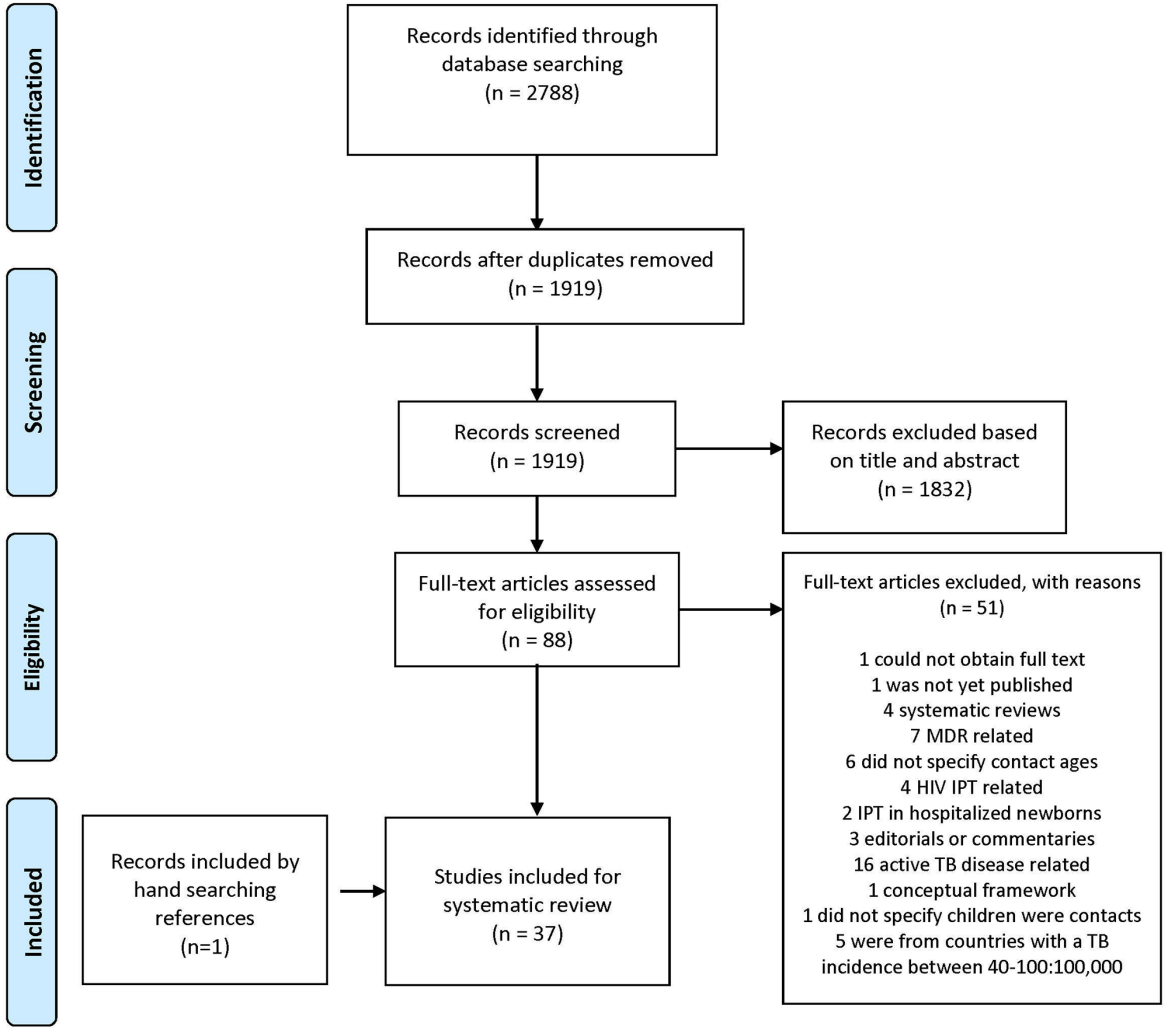
Study selection.

Our initial search was performed between January 1, 1996 and October 25, 2016 and was updated to include articles through May 23, 2017. After removal of duplicate entries, 1919 studies were evaluated using titles and abstracts; 87 full text studies were reviewed for eligibility and 36 studies were included in the systematic review. Hand searching by reviewing all references of included studies yielded one additional study. In total, 37 studies were included in the systematic review (Fig 2).

### Study characteristics

Table 1 summarizes characteristics of the 37 studies included in the review [21, 23, 26–60]. Of 37 studies, 22 studies were conducted in the African region, 14 in Southeast Asia, and one in the Americas (Peru). The countries where most studies originated in the Africa region were South Africa (n = 10), Ethiopia (n = 4), and Malawi (n = 3); and in the Southeast Asia region were India (n = 5) and Indonesia (n = 4). Most studies were published in the last eight years (2009–2017) with no studies published in the first five years (1997–2001) and only six studies published in the five years between 2002 and 2006. Most studies (n = 25) conducted were quantitative evaluations, three studies were qualitative and a growing number (n = 9) incorporated mixed methods. Of quantitative or mixed studies, 19 of 34 (56%) pulled data from program settings and operational definitions throughout the CCM cascade varied greatly.

**Table 1:** Detailed characteristics of studies included in review.

Author	Title of Study	Pub Date	Journal	Study Year	Type of Study	Country	TB Incidence 2015	# of CCs identified <5 yrs	# of CCs screened <5 yrs	% Screened	# of CCs <5 yrs eligible for IPT	% Eligible	# of CCs initia-ting treat-ment <5yrs	% Initiated	# of CCs com-pleting treat-ment <5 yrs	% Completed^3^
**AFRICA**																
Arscott-Mills, T	Survey of health care worker knowledge about childhood tuberculosis in high-burden centers in Botswana	2017	IJTLD	2012	Qualitative	Botswana	356	N/A	N/A	N/A	N/A	N/A	N/A	N/A	N/A	N/A
Assefa, D	Cross sectional study evaluating routine contact investigation in Addis Ababa, Ethiopia: A missed opportunity to prevent tuberculosis in children	2015	PLoS One	2013	Mixed methods	Ethiopia	192	230	78	33.9%	76	97.4%	3	3.9%	N/R	N/R
Chabala, C	Missed opportunities for screening child contacts of smear-positive TB in Zambia, a high-prevalence setting	2017	IJTLD	2013	Quantitative	Zambia	391	273	N/R	N/R	N/R	N/R	N/R	N/R	N/R	N/R
Claessens, NJM	Screening childhood contacts of patients with smear-positive pulmonary tuberculosis in Malawi	2002	IJTLD	2001	Quantitative	Malawi	193	365	33	9.0%	27	81.8%	23	85.2%	N/R	N/R
Egere,	Isoniazid preventive treatment among child contacts of adults with smear-positive tuberculosis in The Gambia	2016	PHA	2013–2015	Quantitative	Gambia	174	404	404	100.0%	368	91.1%	328	89.1%	310	94.5%
Garie, KT	Lack of adherence to isoniazid chemoprophylaxis in children in contact with adults with tuberculosis in Southern Ethiopia	2011	PLoS One	2007–2009	Quantitative	Ethiopia	192	82	82	N/R^4^	82	100.0%	82	100.0%	10	12.2%
Gomes, VF	Adherence to isoniazid preventive therapy in children exposed to tuberculosis: a prospective study from Guinea-Bissau	2011	IJTLD	2005–2007	Quantitative	Guinea-Bissau	373	N/R^1^	736	N/R	N/R	N/R	609	82.7%^2^	N/R	N/R
Marais, BJ	Adherence to isoniazid preventive chemotherapy: a prospective community based study	2006	Arch Dis Child	2003–2005	Quantitative	South Africa	834	274	229^5^	83.6%	N/R	N/R	180	N/R	36	20.0%
Nyirenda, M	Poor attendance at a child TB contact clinic in Malawi	2006	IJTLD	2003–2005	Quantitative	Malawi	193	N/R	N/R	N/R	N/R	N/R	N/R	N/R	N/R	N/R
Osman, M	Routine programmatic delivery of isoniazid preventive therapy to children in Cape Town, South Africa	2013	PHA	2010	Quantitative	South Africa	834	525	244	46.5%	N/R	N/R	141	57.8%^2^	19	13.5%
Ramos, JM	Screening for tuberculosis in family and household contacts in rural area in Ethiopia over a 20-month period	2013	IJMyco	2011–2012	Quantitative	Ethiopia	192	N/R	34	N/R	N/R	N/R	22	64.7%^2^	N/R	N/R
Skinner, D	Pasting together the preventive therapy puzzle	2013	IJTLD	2012	Quantitative	South Africa	834	N/R	N/R	N/R	N/R	N/R	N/R	N/R	N/R	N/R
Skinner, D	It’s hard work, but it’s worth it: the task of keeping children adherent to isoniazid preventive therapy	2013	PHA	2011	Qualitative	South Africa	834	N/A	N/A	N/A	N/A	N/A	N/A	N/A	N/A	N/A
Szkwarko, D	Implementing a tuberculosis child contact register to quantify children at risk for tuberculosis and HIV in Eldoret, Kenya	2013	PHA	2011	Quantitative	Kenya	233	101	N/R	N/R	87	N/R	2	2.3%	N/R	N/R
Tadesse, Y	Uptake of isoniazid preventive therapy among under-five children: TB contact investigation as an entry point	2016	PLoS One	2013–2014	Quantitative	Ethiopia	192	282	237	84.0%	221	93.2%	142	64.3%	114	80.3%
Thind, D	An evaluation of Robolola	2012	IJTLD	2009	Quantitative	South Africa	834	552	361	87.8%^6^	327	N/R	286	87.5%	N/R	N/R
van Soelen, N	Does an isoniazid prophylaxis register improve tuberculosis contact management in South African children?	2013	PLoS One	2008 vs. 2011	Quantitative	South Africa	834	pre-IPT reg: N/R; post-IPT reg: N/R	pre-reg 24; post-reg 39+15 additional entered into IPT reg	pre-reg N/R; post-reg N/R	N/R	N/R	pre-reg 4; post-reg 54	pre-reg 16.7%^2^; post-reg N/R	pre-reg N/R; post-reg 20	pre-reg N/R; post-reg 37.0%
Van Wyk, SS	Recording isoniazid preventive therapy delivery to children: operational challenges	2010	IJTLD	2008	Quantitative	South Africa	834	24	5	N/R^7^	N/R	N/R	4	N/R^7^	N/R	N/R
Van Wyk, SS	Operational challenges in managing isoniazid preventive therapy in child contacts: a high-burden setting perspective	2011	BMC-PH	2008	Quantitative	South Africa	834	149	4	2.7%	149^8^	N/R	2	1.3%	0	0.0%
Van Wyk, SS	TB contact investigation in a high-burden setting: house or household?	2012	IJTLD	2008	Quantitative	South Africa	834	N/R	N/R	N/R	N/R	N/R	N/R	N/R	N/R	N/R
van Zyl, S	Adherence to anti-tuberculosis chemoprophylaxis and treatment in children	2006	IJTLD	1996–2003	Quantitative	South Africa	834	326	301	92.3%	181	60.1%	172	95.0%	29	27.6%
Zachariah, R	Passive versus active tuberculosis case finding and isoniazid preventive therapy among household contacts in rural district of Malawi	2003	IJTLD	2001–2002	Quantitative	Malawi	193	passive: 126^1^; active: 113	passive: 0; active: 44	passive 0%; active 39%	N/R	N/R	passive: 22^9^; active: 25	passive 17.5%^2^; active 22.1%	N/R	N/R
**SOUTH EAST ASIA**																
Amanullah, F	Unmasking childhood tuberculosis in Pakistan: efforts to improve detection and management	2015	IJTLD	2008	Quantitative	Pakistan	270	N/R	256	N/R	N/R	N/R	184	71.9%^2^	60	32.6%
Banu Rekha, VV	Contact screening and chemoprophylaxis in India’s Revised Tuberculosis Control Programme: a situational analysis	2009	IJTLD	2008	Mixed methods	India	217	N/R^1^	84	N/R	84	100.0%	16	19.0%	N/R	N/R
Coprada, L	A review of TB contact investigations in the poor urban areas of Manila, The Philippines	2016	PHA	2012	Mixed methods	Philippines	322	1227	816	66.5%	202	24.8%	200	99.0%	180	90.0%
Hall, C	Challenges to delivery of isoniazid preventive therapy in a cohort of children exposed to tuberculosis in Timor-Leste	2015	TM & IH	2013–2014	Quantitative	Timor-Leste	498	255	66	25.9%	N/R	N/R	46	69.7%^2^	N/R	N/R
Pothukuchi, M	Tuberculosis contact screening and isoniazid preventive therapy in a south Indian district: Operational issues for programmatic consideration	2011	PLoS One	2008	Quantitative	India	217	172^1^	116	67.4%	116	100.0%	97	83.6%	N/R	N/R
Rekha, B	Improving screening and chemoprophylaxis among child contacts in India’s RNTCP: a pilot study	2013	IJTLD	2009–2011	Mixed methods	India	217	87^1^	53	60.9%	53	100.0%	53	100.0%	39	73.6%
Rutherford, M	Adherence to isoniazid preventive therapy in Indonesian children: A quantitative and qualitative investigation	2012	BMC—Res Notes	2009–2010	Mixed methods	Indonesia	395	N/R	N/R	N/R	N/R	N/R	82	N/R	21	25.6%
Rutherford, M	Management of children exposed to Mycombacterium tuberculosis a public health evaluation in West Java Indonesia	2013	Bull of the WHO	2009–2012	Mixed methods	Indonesia	395	N/R	N/R	N/R	cohort 1: 15; cohort 2: N/A	N/R	cohort 1: 6; cohort 2: 82	cohort 1: 40%; cohort 2: N/A	cohort 2: 21	cohort 2: 25.6%
Shivaramakrishna, HR	Isoniazid preventive treatment in children in two districts of South India: does practice follow policy?	2014	IJTLD	2012	Quantitative	India	217	271^1^	218	80.4%	209	95.9%	70	33.5%	16	22.9%
Singh, AR	Isoniazid Preventive therapy among children living with tuberculosis patients: Is it working? A mixed-method study from Bhopal, India	2016	J of Trop Peds	2015	Mixed methods	India	217	59^1^	51	86.4%	50	98.0%	11	22.0%	10	90.9%
Thanh, THT	A household survey on screening practices of household contacts of smear positive tuberculosis patients in Vietnam	2014	BMC-PH	2010	Quantitative	Vietnam	137	293	16	5.5%	N/R	N/R	N/R	N/R	N/R	N/R
Tornee, S	Factors associated with the household contact screening adherence of tuberculosis patients	2005	SE Asian J Trop Med PH	2003	Mixed methods	Thailand	172	N/R	N/R	N/R	N/R	N/R	N/R	N/R	N/R	N/R
Triasih, R	A prospective evaluation of the symptom-based screening approach to the management of children who are contacts of tuberculosis cases	2015	CID	2010–2012	Quantitative	Indonesia	395	N/R	N/R	N/R	N/R	N/R	99	N/R	N/R	50.0%
Triasih, R	A mixed-methods evaluation of adherence to preventive treatment among child tuberculosis contacts in Indonesia	2016	IJTLD	2010–2012	Mixed methods	Indonesia	395	N/R	N/R	N/R	99	N/R	86	86.9%	50	58.1%
**THE AMERICAS**																
Chiang, SS	Barriers to the treatment of childhood TB infection and TB disease: a qualitative study	2017	IJTLD	2012	Qualitative	Peru	119	N/A	N/A	N/A	N/A	N/A	N/A	N/A	N/A	N/A

CCs = Child Contacts, N/A = not applicable, NR = not reported, IJTLD = International Journal of Tuberculosis and Lung Disease, Arch Dis Child = Archives of Disease in Childhood, PHA = Public Health Action, IJMyco = International Journal of Mycobacteriology, Southeast Asian J Trop Med Public Health = The Southeast Asian Journal of Tropical Medicine and Public Health, CID = Clinical Infectious Diseases, Reg = register.

^1^Child contacts < 6 years of age.

^2^When IPT eligibility was not available, IPT initiation was calculated using the number screened as the denominator.

^3^Completion defined as 4 or more months of IPT.

^4^All child contacts identified were screened as part of the study.

^5^Full screening in this study included TST and chest x-ray.

^6^411 used as denominator as 16 child contacts were on TB treatment and 125 were already on PT out of the 552.

^7^Only 5 child folders were found so number of child contacts screened is unknown.

^8^Communication with co-author confirmed that there was no evidence of active TB diagnosis in 149 child contacts identified during retrospective review.

^9^22 child contacts were initiated on IPT by ward nurses without recommended screening.

#### Child contact identification

Of the 34 studies that included quantitative data, 21 included the number of children identified. The sample size of child contacts <5 years identified in these 34 studies varied widely between 24 child contacts in a South African study [39] and 1227 child contacts in a study from the Philippines [58].

#### TB screening

**Fig 3:**
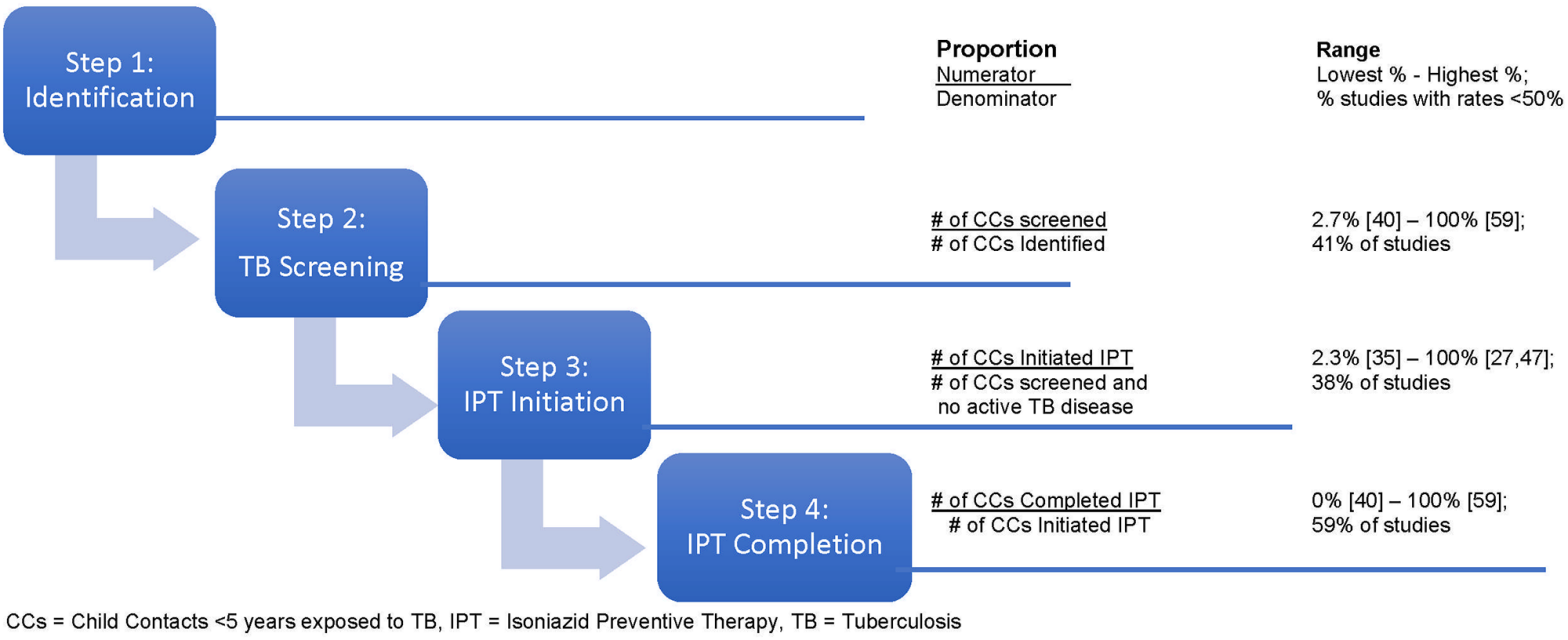
Range of proportions of child contacts completing each CCM cascade step.

In 17 studies that included information regarding TB screening, rates varied between a low of 2.7% of identified child contacts screened in a South African study [40] and a high of 100% screened in a study from the Gambia [59]. In 41% (7/17) of studies, screening rates of <50% were reported (Fig 3).

#### IPT initiation

In 24 studies that included information regarding IPT initiation, rates varied between a low of 2.3% initiation in a Kenyan study [35] and a high of 100% initiation in studies from India [47] and Ethiopia [27]. In 38% (9/24) of studies, initiation rates of <50% were reported (Fig 3).

#### IPT completion

In 17 studies, which included information regarding IPT completion, rates varied between a completion rate of 0% in a South African study [40] to 94.5% completion in a study from the Gambia [59]. In 59% (10/17) of studies, completion rates of <50% were reported. There was one study that measured adherence using a urine test, showing that of those who completed IPT, 255/310 (82%) had good adherence [59] (Fig 3).

### Child TB contact management challenges

A review of the 37 studies revealed many challenges present at every step of the CCM cascade. We categorized these challenges into seven themes and seventeen sub-themes that are summarized in Table 2 and described below.

**Table 2:** Challenges and recommendations.

CHALLENGES	RECOMMENDATIONS
**Health System Infrastructure**	**Health System Strengthening**
Lack of government and NTP prioritization [21, 36, 40, 46, 51, 52]	Introduce monitoring and evaluation tools [21, 23, 28, 35, 36, 38–40, 44, 47, 50, 55, 58, 60]
Lack of tools to support documentation [21, 23, 30, 31, 33, 35, 39–41, 44, 46, 50, 60]	Prioritize CCM and provide support to HCWs and clinics [26, 32, 33, 36, 37, 41, 42, 45–47, 51–53, 55–60]
Limited staff resources [30, 33, 40, 42, 48, 52, 53, 55, 58]	
**Knowledge Gaps**	**Health Education**
Index Case and caregiver education [23, 26, 33, 36, 44, 46, 48, 50, 51, 53, 55–57, 60]	Healthcare worker education [23, 26, 27, 30, 35, 36, 38, 40, 43, 44, 46–48, 50, 52, 53, 55–60]
Healthcare worker education regarding CCM [23, 26, 29–31, 33, 36, 37, 40, 44, 46–50, 53, 55–58, 60]	Index case and caregiver education [23, 27–29, 33, 34, 36, 38, 42, 44, 47, 49, 51–53, 56–59]
Knowledge regarding TB diagnosis [23, 31, 46, 47, 55, 58, 60] Knowledge regarding INH resistance [57]	**Improved Preventive Therapy**
**Attitudes and Perceptions**	Study efficacy and implement shorter regimens [33, 41, 47, 50, 53, 54]
Risk Perception* [23, 27–30, 33, 38, 42, 47–49, 51–53, 55, 57, 60]	Synchronize IC and CC visits [42]
Patient-provider relationship [33, 58]	Ensure availability of IPT [23, 27, 46, 60] Create child friendly formulations [49, 54, 55, 57, 60]
**Stigma**	
Stigma [21, 33, 34, 38, 55]	
**Access to Care**	
Cost, including transport, screening, diagnostic testing, and treatment [23, 30, 35, 42, 45, 48, 49, 51–53, 58]	
Travel time and coordination [23, 28, 43, 45, 47–49, 52, 55, 59]	
Wait times and clinic schedule [23, 33, 42, 48, 53]	
**Competing Priorities**	
Family priorities [33, 42, 47, 48, 51, 52, 58, 59]	
**Treatment Related**	
Medication, including size, taste, duration of treatment [48–50, 53, 60]	
Experienced side effects [47–49]	
Ability to administer treatment to child contact [28, 33, 57, 59]	
INH Procurement [23, 50, 58, 60]	

CCM = Child TB Contact Management, IC = Index Case, CC = Child Contact, HCW = Healthcare Worker, NTP = National TB Program.

* Caregivers’ perceived low risk if child was healthy and asymptomatic.

#### Health system infrastructure

Health system infrastructure challenges could be divided into three categories: lack of government and NTP prioritization, lack of tools to support documentation, management, and monitoring and evaluation (M&E) of CCM, and limited resources. At the government level, lack of NTPs’ leadership to prioritize CCM implementation was reported (n = 6). Heavy demands to care for large numbers of TB cases, including TB-HIV co-infection cases, were reported as reasons for poor performance of CCM strategies. Additionally, at the clinic level, studies identified lack of tools to support documentation throughout CCM as a barrier (n = 13). This included lack of documents to record child contacts as they move through every step of the CCM cascade. Although some programs reported having a system or some tools in place for recording and reporting, these are not standardized across facilities and insufficient for effective program monitoring and evaluation of CCM. Finally, limited staff resources were identified as a barrier (n = 9). CCM increases HCW workload with additional monthly visits for child contacts. One study reported that prioritizing index cases over child contacts was detrimental to CCM in a setting with limited staff [40].

#### Knowledge gaps

Challenges arising from inadequate knowledge of index cases, caregivers and HCWs were identified. Index cases and caregivers did not receive adequate health education or did not comprehend information regarding CCM (n = 14). In particular, caregivers were uninformed about the need for child contact screening and the importance of IPT. For example, two studies reported that few index cases were informed about screening child contacts (21% from Malawi, 32% from Zambia) [26, 56]. Several studies mentioned challenges with knowledge regarding IPT benefits and duration of therapy, which led to non-initiation and non-adherence.

HCW knowledge gaps, lack of guidelines, and non-adherence to guidelines were found in many studies (n = 21). An Indonesian healthcare worker said, “My understanding of IPT is limited. There is no guideline for IPT available. I have no confidence to explain IPT to the patient.”[53] Several studies mentioned that HCWs experienced challenges in ruling out TB disease due to unavailability of tuberculin skin testing and/or chest x-ray, inability to interpret these results, and overall lack of knowledge regarding the screening necessary to rule out TB disease and initiation of IPT. One study reported that health center personnel were concerned that IPT leads to INH resistance [57].

#### Attitudes and perceptions

Attitudes and perceptions about the need for screening and IPT presented important challenges. Caregivers were reluctant to have child contacts screened or initiated on IPT despite education regarding the importance of TB prevention (n = 17). The most common reason was that child contacts were healthy and asymptomatic, and therefore caregivers did not see a need for TB screening and/or IPT. An Indonesian caregiver said “I wasn’t concentrating on that [IPT] because I thought [the child] was healthy anyway” [49]. Two studies noted that patient/provider relationship can also influence IPT adherence, with negative relationships potentially hampering adherence [33, 58].

#### Stigma

Another important concern was stigma. Caregivers felt that child contact screening could lead to unwanted disclosure of TB and/or HIV to other family members and neighbors (n = 5). As TB remains associated with HIV, IPT could be perceived as a marker of HIV in addition to TB. A parent in a South African study stated “Most of people who have TB…feel they would rather stay home because if they go to the clinic they will meet so and so who will start gossiping about them. They will label me as HIV as I have seen done to others. In the end children end up not getting treatment” [33].

#### Access to care

A major challenge identified by studies (n = 17) was the ability of caregivers and their child contacts to access care. This was found to be a challenge that affected every CCM cascade step. Caregivers struggled to bring child contacts to the clinic for TB screening and IPT provision. The most common challenge discussed was cost, including cost of transport, screening, diagnostic testing, and medications (n = 1). A second major challenge was transportation barriers, including long journey times to clinics and migration of child contacts for reasons such as living with extended family (n = 10). Finally, long wait times and inconvenient clinic hours for working caregivers were reported in several studies as an access to care barrier (n = 5); one study reported wait times of up to four hours [53].

#### Competing priorities

Another major barrier to child contact screening and adherence reported was family prioritization of other competing needs over IPT (n = 8). Studies reported a variety of such other priorities (i.e. parents’ work schedules, child contacts’ need to go to school). Some studies reported that the relationship of the index case and the child contact impacted the prioritization of CCM (n = 3). Specifically, non-parent index cases may not pass information on to the primary caregivers and caregivers therefore may not bring child contacts in for screening [45]. One study reported that index cases felt that if they were not directly taking care of their contacts, the children had a low risk of getting TB [52]. Finally, one study reported that the death or nonadherence of the index case led to challenges to retain child contacts in care [47].

#### Treatment-related challenges

Treatment-related issues were found to be important barriers to adherence (n = 12). Size of pills, bitter taste, and long duration of treatment were reported (n = 5), as were perceived medication side effects (n = 3). Administering isoniazid (INH) to the child contact was a problem for some caregivers (n = 4); for example, one study reported a concern that the parents may not see their children every day to be able to give them their medication [33] or that they may forget to give the medication [28, 59]. Some studies reported that according to HCWs, procurement of INH may be a barrier to CCM (n = 4).

### Child TB contact management recommendations

The 37 studies included in the review incorporated recommendations to improve CCM; these recommendations were categorized into three themes that are summarized in Table 2 and described below.

#### Health system strengthening

Most studies included health system strengthening recommendations (n = 28). One major recommendation made in many studies, was to create and introduce M&E tools for CCM (n = 14), which included separate child contact registers, IPT cards, and IPT registers. Another recommendation was that NTPs need to prioritize CCM and provide support to HCWs and TB clinics (n = 19). One study suggested quarterly supervisory visits with routine measurement of how well CCM has been implemented [26]. Another study suggested that HCWs coordinate with other agencies like daycare centres or schools to conduct screening of child contacts [58]. A third study included recommendations from nursing staff who suggested applying a DOTs approach in the first few months of treatment to improve caregiver adherence [33].

#### Health education

Many studies recommended health education for HCW’s (n = 22) as well as index cases, caregivers, and the community (n = 19). Studies recommended that HCW’s competence be improved through training, mentorship, program monitoring, and supportive supervision. Specifically, it was recommended that HCWs be adequately trained regarding the importance of contact screening so that they can effectively pass on this information to the community. Furthermore, effective education and communication packages for caregivers were seen as essential tools for HCWs to address caregiver concerns. On the caregiver’s side, most studies suggested continuous health education with the family throughout the six months of IPT. Targeted messages and educational materials to improve families’ awareness of child contact screening were recommended. Additionally, interventions should address the perception that asymptomatic contacts do not need IPT and emphasize the importance of IPT completion. One study also recommended health education regarding infection control so that further spread of TB can be prevented [52].

#### Improved preventive therapy

Fourteen studies recommended improving preventive therapy delivery, which includes studying the efficacy and feasibility of implementing shorter regimens (n = 6), ensuring availability of INH (n = 4), synchronizing index case and child contact visits (n = 1), and creating child friendly formulations (n = 5).

### Predictors of CCM cascade step completion

An analysis of predictors of successful CCM cascade step completion was included in eleven studies. Predictors are summarized by CCM step in Table 3 and described below.

**Table 3:** Predictors of CCM cascade step completion.

Predictors to child contacts completing each CCM step	Comments	Child Contact Ages	Results	Author	Country
*Screening*					
IC is female	Female ICs more likely to bring child contacts than male ICs	<5 years	OR 2.67, p < 0.001	Nyirenda, M [30]	Malawi
IC is parent	Parents more likely to bring child contacts than non-parents	<5 years	OR 2.62, 95% CI 1.46–4.7, p = 0.0013	Hall, C [45]	Timor Leste
Distance or location of TB clinic	Screening at same district better than in other district	<5 years	OR 3.49, 95% CI 1.6–7.66, p = 0.0017	Hall, C [45]	Timor Leste
	Living near clinic better than far from clinic	<15 years	aOR 11.47, 95% CI 4.57–28.79	Tornee, S [52]	Thailand
Perception of susceptibility	High perception of susceptibility to disease better than low	<15 years	aOR 2.90, 95% CI 1.18–7.16	Tornee, S [52]	Thailand
Perception of barriers	Low perception of barriers better than high perception	<15 years	aOR 4.60, 95% CI 1.99–10.60	Tornee, S [52]	Thailand
Intention to bring CC to clinic	Serious intentions better than non-serious intentions	<15 years	aOR 3.35, 95% CI 1.44–7.76	Tornee, S [52]	Thailand
Clinic Location	Urban clinic better than rural clinic	<6 years	72% vs 49%, p = 0.05	Rekha, B [47]	India
		<6 years	RR 6.65, 95% CI 3.06–14.42	Pothukuchi, M [46]*	India
IC shares bedroom	Shares bedroom with any child <15 years of age	<5 years	aOR 2.34, 95% CI 1.18–4.40	Chabala, C [56]	Zambia
HCW provided information	Source of information regarding IPT was from health care provider	<5 years	aOR 3.22, 95% CI 1.11–9.35	Chabala, C [56]	Zambia
IPT Knowledge	IC agreed that IPT should be provided to well children to prevent TB	<5 years	aOR 2.26, 95% CI 1.11–4.60	Chabala, C [56]	Zambia
Facility Type	Non-governmental facility was better than local government unit	<15 years	95.6% vs. 43.5% p< 0.001	Coprada, L [58]	Philippines
*IPT Initiation*					
IC is parent	Parents more likely to initiate child contacts than non-parents	<6 years	46% vs 19%, p = 0.001	Shivaramakrishna, HR [50]	India
	Parents more likely to initiate child contacts than others	<6 years	RR 1.4, 95% CI 1.0–2.0^±^	Singh, AR [60]	India
Home visit	Initial home visit better than no home visit	<6 years	41% vs 17%, p = 0.004	Shivaramakrishna, HR [50]	India
IC shares bedroom	Shares bedroom with any child <15 years of age	<5 years	aOR 4.56, 95% CI 1.53–13.7	Chabala, C [56]	Zambia
IPT Knowledge	IC agreed that IPT should be provided to well children to prevent TB	<5 years	aOR 15.3, 95% CI 1.97–118.9	Chabala, C [56]	Zambia
Distance or location of TB clinic	Child contacts living <5km from public health institution more likely to initiate IPT	<6 years	RR 1.4, 95% CI 1.1–1.6^±^	Singh, AR [60]	India
*IPT Completion*					
Medication costs	Lower medication costs better than higher	<5 years	OR 20, 95% CI 2.7–414.5	Rutherford, M [49]	Indonesia
Transport costs	Lower transport costs better than higher	<5 years	OR 3.3, 95% CI 1.1–10.2	Rutherford, M [49]	Indonesia
Treatment duration	Shorter treatment (3HR) better than longer treatment (6H)	<5 years	67% vs 27%, OR 4.97, 95% CI 2.4–10.36, p< 0.001	Van Zyl, S [41]	South Africa
Supervision	Supervision by HCW or community supporter is better than supervision by caregiver/index case	<5 years	OR 4.43, 95% CI 1.47–13.72, p = 0.006	Van Zyl, S [41]	South Africa
Clinic location	Rural clinic better than urban clinic	<6 years	95% vs 61%, p< 0.01	Rekha, B [47]	India

*Screening and IPT initiation were analyzed together.

^±^Relative risk was calculated with variables “distance > 5km vs. 5-10km” and “child lives with other individual vs. parent” and outcome “not initiating IPT.”

CCM = Child TB Contact Management, IC = Index Case, CC = Child Contact, HCW = Healthcare Worker, OR = Odds Ratio, CI = Confidence Interval, aOR = Adjusted Odds Ratio, RR = Relative Risk.

#### Screening

Predictors that were significantly associated with child contacts being brought in for screening included: the index case was a parent, the index case was female, the child contact lived near the TB clinic, the index case had a high perception of child contact susceptibility to disease, the index case had a low perception of barriers to treatment, the index case had serious intention to bring the child in for screening, the child contact was identified at an urban clinic rather than a rural clinic, the index case reported sharing a bedroom with any child <15 years, information regarding IPT was provided to the index case by a HCW, index case agreed that IPT should be given to well children to prevent TB, and facility was non-governmental rather than a local governmental unit [30, 45–47, 52, 56, 58].

#### IPT initiation

Predictors that were significantly associated with child contacts initiating IPT included: the index case was a parent, child contact had a home visit, index case reported sharing a bedroom with any child <15 years, index case agreed that IPT should be given to well children to prevent TB, and distance <5km from the public health institution [50, 56, 60].

#### IPT completion

Predictors that were significantly associated with IPT completion included: low medication costs, low transport costs, shorter regimen, directly observed therapy by HCW or community supporter, and initiating therapy at a rural site [41, 47, 49].

## Discussion

This is the first systematic review to examine the CCM cascade in HBCs with the goal of providing guidance to NTPs that are establishing or improving CCM. We used mixed-methods to report child contact losses at each CCM cascade step, to identify CCM strategies implemented in HBCs, and to summarize common challenges and positive predictors of CCM outcomes. We found many losses at each CCM cascade step, with variability in the definitions that programs used throughout the CCM cascade. Additionally, we found great variation in the rates of TB identification, screening, IPT initiation, and IPT completion. While a few studies reported high IPT initiation and completion rates, most programs struggled with suboptimal CCM.

The first WHO guidelines to recommend CCM were published in 2006. Therefore, not surprisingly, most studies included in the review were published in the last eight years despite searching since 1996. This likely represents an important shift in CCM after publication of the WHO guidelines with more countries realizing the potential impact of CCM to prevent TB amongst the young and vulnerable. Interestingly, almost a third of studies (10/37) were from South Africa, reporting the highest TB incidence in the world in 2015 [1].

**Fig 4:**
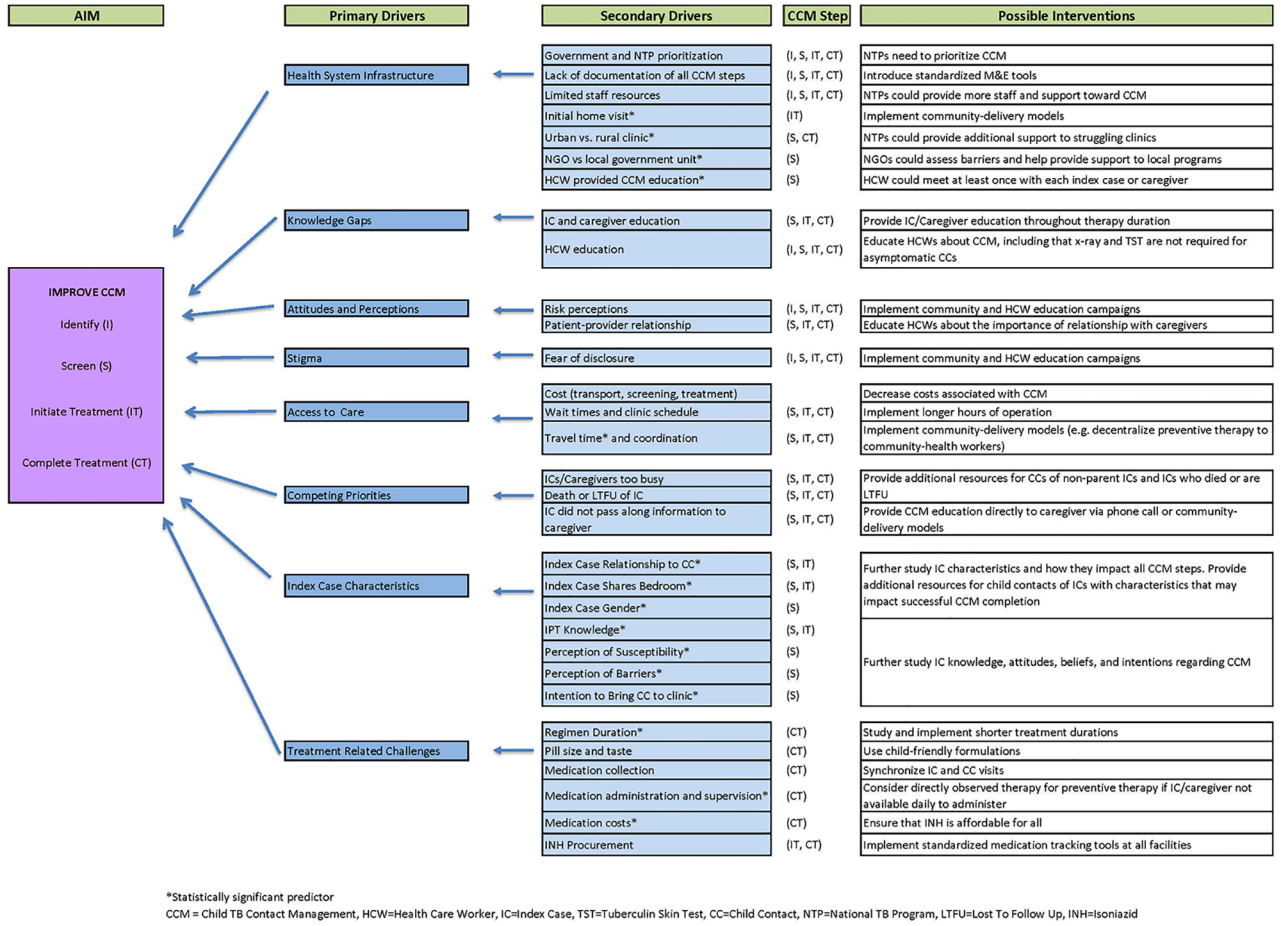
Driver diagram for child contact management.

We used the HIV care cascade as a model for the CCM cascade, which provided a framework to separate the primary and secondary drivers impacting each of the four cascade steps. Ultimately, the aim for CCM in HBCs is to (1) identify all child contacts exposed to a bacteriologically confirmed TB case, (2) screen all identified child contacts for active TB disease, (3) ensure that all eligible screened contacts either initiate treatment for active TB or IPT, and (4) support treatment completion. Applying the HIV care cascade to TB infection has been successfully utilized by other researchers in a broader population [17]. Based on our results, we compiled a driver diagram (Fig 4) to provide further guidance to NTPs and the research community on improving CCM. The challenges and predictors identified translate into the primary drivers that need to be addressed to achieve successful CCM. These include: health system infrastructure, knowledge gaps, attitudes and perceptions regarding CCM, stigma, access to care, competing priorities, index case characteristics, and treatment related challenges.

Health system infrastructure was identified as a primary driver that impacts all four steps in the CCM cascade. The secondary drivers for health system infrastructure include government and NTP prioritization of CCM, limited staff resources, and lack of documentation of all steps, facility type (urban vs. rural, NGO vs. government), and type of HCW conducting CCM education. With the recent requirement by the WHO to report IPT initiation in child contacts <5 years, political will to implement and improve CCM will likely increase from the ministry level. Prioritization of CCM by NTPs has the greatest potential impact, as this driver will ultimately influence all other primary and secondary drivers.

An important aspect of CCM prioritization by NTPs will be selecting implementation or intervention strategies to optimize all CCM cascade steps. Both passive and active approaches to CCM have been reported. Passive approaches include methods such as HCWs verbally inviting index cases to bring child contacts for screening without follow up when contacts are not brought in. Some studies included in our review incorporated active CCM strategies such as the implementation of: home visits for identification and screening, M&E tools that document all steps of CCM (clinic-based), and medication administration (community-based). It is unclear which of these strategies are most effective, and further research to compare effectiveness of different CCM approaches (e.g. clinic-based vs community-based) as well as cost analyses are needed.

Although frequently recommended, standard M&E tools for CCM are generally lacking. Most studies that recommended M&E focused on IPT initiation and completion tools. Several studies that incorporated active strategies to track child contacts reported improved identification rates as well as high screening rates after implementation of an IPT register. A WHO recommended standardized CCM register that incorporates all four CCM cascade steps would likely be an effective and efficient tool. Tools should also incorporate INH supply tracking to prompt medication ordering and prevent clinic stock-outs. With the new WHO IPT reporting requirement, standardized tools may assist NTPs in reporting this outcome.

Another primary driver impacting all four CCM steps is knowledge gaps of HCWs as well as index cases and caregivers. Providing CCM support to clinics from the ministry level in order to incorporate standardized education for HCWs is an important component that could also result in improved health education provided to index cases and caregivers. Health education strategies should ideally be longitudinal, available on demand, and practice-based in order to provide ongoing CCM support. With rapid technological advances and widespread mobile networks in HBCs, using mobile messaging programs such as WhatsApp Messenger [43] or utilizing videoconferencing like the ECHO model [61] could enable NTPs to provide continuous education to rural areas at minimal cost.

Several studies reported the inability of HCWs to rule out active TB disease during the CCM screening step when TSTs and x-rays are lacking in clinics. In 24 studies in this systematic review that reported data on IPT initiation in child contacts screened, 9 (38%) reported that fewer than 50% of child contacts screened were initiated on IPT. There are two main programmatic scenarios during which children may get lost in the screening to IPT initiation cascade step: (1) Children who screen positive on the symptom-based screening and are referred to higher levels of care for additional diagnostics, and (2) All child contacts (including asymptomatic) who reside in a country where NTP guidelines require tests such as x-ray or TST prior to initiating IPT and these tests are unavailable at the community level therefore prompting referral to higher level facilities.

Diagnosing TB and obtaining bacteriologic confirmation in children remains a challenge globally which further complicates CCM. The difference between the difficult task of obtaining bacteriologic confirmation in a child with presumptive TB disease and ruling out TB disease in an asymptomatic child contact to initiate IPT should not be confused with one another. A symptom-based screening approach to rule out TB disease in child contacts and initiate IPT is recommended by the WHO [9, 11]. As demonstrated by Triasih and colleagues [54], symptom-based screening is a simple and safe screening method, and special investigations for identifying or excluding TB disease can be reserved for symptomatic children [62]. Children who screen symptomatic on symptom-based screening, and are then referred to higher levels of care for TB diagnostics are at great risk of being lost, particularly those in whom TB disease is excluded and are told to return to the community level for IPT initiation. Implementing a symptom-based screening approach, incorporating HCW education to reinforce the adequacy of symptom-based screening to rule out active TB for CCM [54], and improving communication between community and higher level facilities are key steps in decreasing the losses between screening to rule out TB disease and IPT initiation.

In addition to improving HCW education regarding screening, there are knowledge gaps regarding INH resistance. One study suggested that IPT initiation may be hindered by HCW concerns that IPT can result in the development of INH-resistant TB [59]. Although not reported in other studies included in this review, in our experience, this is a concern that is frequently discussed among HCWs working in CCM. There is no evidence that IPT will result in the development of resistance in children, therefore, this also highlights the need for evidence based education of HCWs by NTPs [63].

Attitudes and perceptions about CCM constitute a primary driver that impacts the entire cascade. Despite receiving health education, index cases and caregivers in many studies were reluctant to bring their child contacts for screening or initiate IPT as they felt that they were healthy and asymptomatic. In many cultures, people do not endorse preventive treatments and there is a misperception that they do not need to take medicines when not feeling sick.

Some of the attitudes and perceptions about CCM may also be closely intertwined and perpetuate another primary driver–stigma. CCM stigma is likely linked with historic TB stigma as well as HIV stigma in settings with high TB-HIV co-infection rates. Index cases may not have disclosed their TB status to families so bringing child contacts in for screening could potentially risk disclosure. Furthermore, HCWs who live in the same communities as patients may sympathize with patients regarding TB disclosure–their efforts to protect them may actually hinder CCM activities. Although not found in our systematic review, we suspect that HCW stigma regarding CCM may be a secondary driver influencing all cascade steps. Continued sensitization of HCWs and communities through population based health education/TB campaigns would likely increase knowledge, reduce stigma, and change attitudes and perceptions regarding CCM.

Access to care, competing priorities, and index case characteristics are drivers that impact screening, IPT initiation, and IPT completion. Secondary drivers such as transport cost and clinic wait times could ideally be prioritized from the ministry level downward. Solutions may include decentralizing and expanding CCM capacity such as offering active CCM strategies, decreasing costs for CCM, and increasing staff and clinic capacity to decrease wait times. Competing priorities such as conflicting caregiver and child contact schedules and index case characteristics may be harder to address but could be reduced by enhancing patient-centered care in TB facilities and allowing the caregivers of children to be involved in creating a more flexible care-plan for their situation.

Most HBCs are also low-resource settings where families struggle financially and often face catastropic costs due to TB, especially if the caregiver is co-infected with HIV [19]. Hence, economic barriers often prevent caregivers from bringing child contacts to the clinic or initiating IPT. Strategies that minimize costs while affording flexibility to caregivers will likely make it easier for families to access and complete IPT. Possible solutions include: investing more time in family discussions to devise a family centered plan before IPT initiation, identifying someone else to bring the child for follow-up visits if the primary caregiver is not available, or shifting to community-based delivery of IPT. Index case characteristics such as gender, relationship to child contact, and index case knowledge regarding IPT are important predictors that warrant further study to identify child contacts needing additional support.

Treatment-related challenges including regimen duration; pill size and taste; medication collection; administration and supervision; medication costs; and INH procurement, constitute a primary driver that impacts IPT initiation and completion. Recently, shorter regimens such as three months of a once-weekly combination of rifapentine and isoniazid have been shown to be safe and effective in children [64]. Implementing shorter regimens would likely improve adherence. Child-friendly fixed dose combinations that are dissolvable and taste good have been available for active TB treatment as of 2016 [65, 66]. Similar formulations for preventive therapy are needed and will likely be better tolerated. Synchronizing follow up visits for the child contact and the index case is a strategy to consider for improving treatment completion. As Egere and colleagues demonstrated, incorporating urine testing for adherence may help programs evaluate their medication monitoring strategies [59]. Finally, ensuring a reliable supply of INH is essential as drug shortages disrupt IPT.

Our review demonstrates that there are many factors related to the successful delivery of preventive therapy among child household contacts, however, the broad structure to support effective and comprehensive CCM is often lacking in HBCs. Pillar one in the End TB Strategy urges us to put patients at the heart of service delivery and create an integrated, patient-centered care and prevention approach [18, 19]. This requires a robust paradigm shift toward prevention and family-based TB care in order to promote more equitable distribution of resource allocation and support the creation of a CCM friendly healthcare environment. From our review, we recommend that a standardized guideline for an effective CCM strategy should include the following: (1) active strategy to identify contacts at risk, (2) structure, processes and tools in facilities and community to implement, track, and monitor CCM, (3) ongoing training and education for HCWs tasked with implementing CCM, (4) tools and strategies to educate caregivers, index patients and the community as a whole, and (5) research toward improved, shorter and child-friendly preventive therapy treatment regimens. Using this structure, programs can perform additional qualitative research and customize interventions to address CCM barriers in ways that are context specific and appropriate.

Our review has several limitations. Most studies did not uniformly report on the CCM cascade steps making it difficult to compare the losses at different CCM steps between studies. For example, some studies reported the number of index cases bringing child contacts for screening rather than the number of child contacts screened. It was not possible to incorporate these results in our review since index cases may have brought in multiple children <5 years for screening. However, standardization of CCM reporting seems to be improving in more recent studies. Nonetheless, the heterogeneity of study designs employed to examine contact tracing limited our ability to conduct a meta-analysis and calculate summary estimates. Second, we noted inconsistencies in CCM definitions. There was an inconsistency between reporting child contacts <5 years versus <6 years; seven of 37 studies reported data for child contacts aged <6 years. With the recent requirement by the WHO to report IPT initiation outcomes in child contacts <5 years, we anticipate future uniformity that will allow for better quantitative analysis of the CCM cascade. Studies did not always report their definition of a household. Household definition determines the pool of identified child contacts who will be tracked throughout the CCM cascade. Further research to study and standardize this definition is needed and we encourage investigators to include their household definitions in future literature. Similarly, the definition of IPT eligibility varies and we encourage programs to look closely at IPT deferment reasons. Many deferred child contacts are also missed opportunities between the screening and IPT initiation phase. Third, given the programmatic nature of CCM, it is likely that work was presented at conferences but has not been published in the literature. We did not include conference proceedings or other grey literature in the review, which could have caused a publication bias. Finally, the challenges and recommendations in our review are a compilation of qualitative data as well as authors’ observations and programmatic experiences. The latter have not been formally studied and more research is needed to improve global generalizability.

## Conclusions

Despite a decade-long recommendation to conduct CCM in HBCs, many child contacts are still lost at each step of the CCM cascade. Our review reveals that HBCs experience similar challenges and implementation gaps globally in conducting CCM. Prioritization of a CCM friendly healthcare environment is imperative and should include structure, processes and tools to implement and monitor CCM; health education interventions for HCWs, caregivers, index cases and the community; and active, evidence-based strategies to eliminate barriers. A focused approach toward every aspect of the CCM cascade will likely diminish losses throughout the CCM cascade and ultimately decrease TB related morbidity and mortality in children.

## Supporting information

S1 File Search strategies for electronic databases. (DOCX)

S2 File PRISMA checklist. (DOC)

S3 File CCM dataset July 2017. (XLSB)
