# Supplementary material for: Child contact management in high tuberculosis burden countries: A mixed-methods systematic review
Source: PLoS One. 2017 Aug 1;12(8):e0182185. doi: 10.1371/journal.pone.0182185 (PMC5538653; doi:10.1371/journal.pone.0182185)
Supplement: S1 File — (DOCX) [file pone.0182185.s001.docx]

**S1 File. Search strategies for electronic databases**

**PubMed/MEDLINE (NCBI)**

1375 records returned on October 25, 2016

Limited to English and date range 1996/01/01 - 2016/10/25

The search was rerun on 5/23/2017 and 26 new articles were discovered.

**Total = 1401 records**

("Tuberculosis"[MeSH] OR Tuberculosis[tiab] OR Tuberculoses[tiab] OR "Latent Tuberculosis"[MeSH] OR Latent Tuberculosis[tiab] OR Latent Tuberculosis Infection[tiab] OR Latent Tuberculosis Infections[tiab] OR "Mycobacterium tuberculosis"[MeSH] OR Mycobacterium tuberculosis[tiab])

 AND

 (Isoniazid preventive therapy[tiab] OR Isoniazid preventive treatment[tiab] OR Isoniazid prophylaxis[tiab] OR Isoniazid prophylactic therapy[tiab] OR Isoniazid treatment[tiab] OR Isoniazid therapy[tiab] OR "Chemoprevention"[MeSH] OR Chemoprevention[tiab] OR chemoprophylaxis[tiab] OR Contact management[tiab] OR Contact screening[tiab] OR "contact tracing"[MeSH] OR contact tracing[tiab] OR "prevention and control"[subheading] OR preventive therapy[tiab] OR prophylaxis[tiab] OR prevention[tiab])

 AND

 ("Child"[MeSH] OR Child[tiab] OR "Child, Preschool"[MeSH] OR "infant"[MeSH] OR infant[tiab] OR "Adolescent"[MeSH] OR adolescent[tiab] OR pediatric[tiab])

 AND

 ("Brunei Darussalam" OR Cambodia OR China OR Taiwan OR Fiji OR Guam OR "Hong Kong" OR Kiribati OR "Republic of South Korea" OR "South Korea" OR Laos OR Macau OR Malaysia OR "Marshall Islands" OR Micronesia OR Mongolia OR Nauru OR "Northern Mariana Islands" OR Palau OR "Papua New Guinea" OR Philippines OR Singapore OR "Solomon Islands" OR Tuvalu OR Vanuatu OR Vietnam OR Bangladesh OR Bhutan OR Burma OR "East Timor" OR India OR Indonesia OR "Democratic People’s Republic of Korea" OR Korea OR Maldives OR Nepal OR "Sri Lanka" OR Thailand OR Afghanistan OR Djibouti OR Iraq OR Libya OR Morocco OR Pakistan OR Somalia OR Sudan OR Yemen OR Algeria OR Angola OR Benin OR Botswana OR "Burkina Faso" OR Burundi OR Cameroon OR "Cape Verde" OR "Central African Republic" OR Chad OR Congo OR "Democratic Republic of Congo" OR "Ivory Coast" OR "Equatorial Guinea" OR Ethiopia OR Eritrea OR Gabon OR Gambia OR Ghana OR Guinea OR "Guinea-Bissau" OR Kenya OR Lesotho OR Liberia OR Madagascar OR Malawi OR Mali OR Mauritania OR Mozambique OR Namibia OR Niger OR Nigeria OR Rwanda OR "Sao Tome and Principe" OR Senegal OR "Sierra Leone" OR "South Africa" OR "South Sudan" OR Swaziland OR Tanzania OR Togo OR Uganda OR Zambia OR Zimbabwe OR Armenia OR Azerbaijan OR Belarus OR "Bosnia and Herzegovina" OR Georgia OR Greenland OR Kazakhstan OR Kyrgyzstan OR Latvia OR Lithuania OR Moldova OR Romania OR Russia OR Tajikistan OR Turkmenistan OR Ukraine OR Uzbekistan OR Bolivia OR Brazil OR "Dominican Republic" OR Ecuador OR "El Salvador" OR Guatemala OR Guyana OR Haiti OR Honduras OR Nicaragua OR Panama OR Paraguay OR Peru)

**SCOPUS (Elsevier)**

924 records returned on October 25, 2016

Limited to English and date range 1996-2016

The search was rerun on 5/23/2017 and 26 new articles were discovered.

**Total = 950 records**

TITLE-ABS-KEY (Tuberculosis OR Tuberculoses OR "Latent Tuberculosis" OR "Latent Tuberculosis Infection" OR "Latent Tuberculosis Infections" OR "Mycobacterium tuberculosis")

AND

TITLE-ABS-KEY ("Isoniazid preventive therapy" OR "Isoniazid preventive treatment" OR "Isoniazid prophylaxis" OR "Isoniazid prophylactic therapy" OR "Isoniazid treatment" OR "Isoniazid therapy" OR Chemoprevention OR chemoprophylaxis OR "Contact management" OR "Contact screening" OR "contact tracing" OR "prevention and control" OR "preventive therapy" OR prophylaxis OR prevention) AND

TITLE-ABS-KEY (Child OR "Preschool Child" OR infant OR adolescent OR pediatric)

AND

TITLE-ABS-KEY ("Brunei Darussalam" OR Cambodia OR China OR Taiwan OR Fiji OR Guam OR "Hong Kong" OR Kiribati OR "Republic of South Korea" OR "South Korea" OR Laos OR Macau OR Malaysia OR "Marshall Islands" OR Micronesia OR Mongolia OR Nauru OR "Northern Mariana Islands" OR Palau OR "Papua New Guinea" OR Philippines OR Singapore OR "Solomon Islands" OR Tuvalu OR Vanuatu OR Vietnam OR Bangladesh OR Bhutan OR Burma OR "East Timor" OR India OR Indonesia OR "Democratic People’s Republic of Korea" OR Korea OR Maldives OR Nepal OR "Sri Lanka" OR Thailand OR Afghanistan OR Djibouti OR Iraq OR Libya OR Morocco OR Pakistan OR Somalia OR Sudan OR Yemen OR Algeria OR Angola OR Benin OR Botswana OR "Burkina Faso" OR Burundi OR Cameroon OR "Cape Verde" OR "Central African Republic" OR Chad OR Congo OR "Democratic Republic of Congo" OR "Ivory Coast" OR "Equatorial Guinea" OR Ethiopia OR Eritrea OR Gabon OR Gambia OR Ghana OR Guinea OR "Guinea-Bissau" OR Kenya OR Lesotho OR Liberia OR Madagascar OR Malawi OR Mali OR Mauritania OR Mozambique OR Namibia OR Niger OR Nigeria OR Rwanda OR "Sao Tome and Principe" OR Senegal OR "Sierra Leone" OR "South Africa" OR "South Sudan" OR Swaziland OR Tanzania OR Togo OR Uganda OR Zambia OR Zimbabwe OR Armenia OR Azerbaijan OR Belarus OR "Bosnia and Herzegovina" OR Georgia OR Greenland OR Kazakhstan OR Kyrgyzstan OR Latvia OR Lithuania OR Moldova OR Romania OR Russia OR Tajikistan OR Turkmenistan OR Ukraine OR Uzbekistan OR Bolivia OR Brazil OR "Dominican Republic" OR Ecuador OR "El Salvador" OR Guatemala OR Guyana OR Haiti OR Honduras OR Nicaragua OR Panama OR Paraguay OR Peru)

(PUBYEAR > 1995)

**Web of Science (Thomson Reuters)**

416 records returned on October 25, 2016

Limited to English and time span 1996-2016

The search was rerun on 5/23/2017 and 21 new articles were discovered.

**Total = 437 records**

(Tuberculosis OR Tuberculoses OR "Latent Tuberculosis" OR "Latent Tuberculosis Infection" OR "Latent Tuberculosis Infections" OR "Mycobacterium tuberculosis")

AND

("Isoniazid preventive therapy" OR "Isoniazid preventive treatment" OR "Isoniazid prophylaxis" OR "Isoniazid prophylactic therapy" OR "Isoniazid treatment" OR "Isoniazid therapy" OR Chemoprevention OR chemoprophylaxis OR "Contact management" OR "Contact screening" OR "contact tracing" OR "prevention and control" OR "preventive therapy" OR prophylaxis OR prevention)

 AND

 (Child OR "Preschool Child" OR infant OR adolescent OR pediatric)

AND

 ("Brunei Darussalam" OR Cambodia OR China OR Taiwan OR Fiji OR Guam OR "Hong Kong" OR Kiribati OR "Republic of South Korea" OR "South Korea" OR Laos OR Macau OR Malaysia OR "Marshall Islands" OR Micronesia OR Mongolia OR Nauru OR "Northern Mariana Islands" OR Palau OR "Papua New Guinea" OR Philippines OR Singapore OR "Solomon Islands" OR Tuvalu OR Vanuatu OR Vietnam OR Bangladesh OR Bhutan OR Burma OR "East Timor" OR India OR Indonesia OR "Democratic People’s Republic of Korea" OR Korea OR Maldives OR Nepal OR "Sri Lanka" OR Thailand OR Afghanistan OR Djibouti OR Iraq OR Libya OR Morocco OR Pakistan OR Somalia OR Sudan OR Yemen OR Algeria OR Angola OR Benin OR Botswana OR "Burkina Faso" OR Burundi OR Cameroon OR "Cape Verde" OR "Central African Republic" OR Chad OR Congo OR "Democratic Republic of Congo" OR "Ivory Coast" OR "Equatorial Guinea" OR Ethiopia OR Eritrea OR Gabon OR Gambia OR Ghana OR Guinea OR "Guinea-Bissau" OR Kenya OR Lesotho OR Liberia OR Madagascar OR Malawi OR Mali OR Mauritania OR Mozambique OR Namibia OR Niger OR Nigeria OR Rwanda OR "Sao Tome and Principe" OR Senegal OR "Sierra Leone" OR "South Africa" OR "South Sudan" OR Swaziland OR Tanzania OR Togo OR Uganda OR Zambia OR Zimbabwe OR Armenia OR Azerbaijan OR Belarus OR "Bosnia and Herzegovina" OR Georgia OR Greenland OR Kazakhstan OR Kyrgyzstan OR Latvia OR Lithuania OR Moldova OR Romania OR Russia OR Tajikistan OR Turkmenistan OR Ukraine OR Uzbekistan OR Bolivia OR Brazil OR "Dominican Republic" OR Ecuador OR "El Salvador" OR Guatemala OR Guyana OR Haiti OR Honduras OR Nicaragua OR Panama OR Paraguay OR Peru)
